# Effect of Forearm Postures and Elbow Joint Angles on Elbow Flexion Torque and Mechanomyography in Neuromuscular Electrical Stimulation of the Biceps Brachii

**DOI:** 10.3390/s23198165

**Published:** 2023-09-29

**Authors:** Raphael Uwamahoro, Kenneth Sundaraj, Farah Shahnaz Feroz

**Affiliations:** 1 Fakulti Kejuruteraan Elektronik dan Kejuruteraan Komputer, Universiti Teknikal Malaysia Melaka, Durian Tunggal 76100, Melaka, Malaysia; raphael.engr@gmail.com (R.U.); shahnaz@utem.edu.my (F.S.F.); 2 Regional Centre of Excellence in Biomedical Engineering and e-Health, University of Rwanda, Kigali P.O. Box 4285, Rwanda

**Keywords:** mechanomyography, electrical stimulation, muscle mechanics, muscle assessment

## Abstract

Neuromuscular electrical stimulation plays a pivotal role in rehabilitating muscle function among individuals with neurological impairment. However, there remains uncertainty regarding whether the muscle’s response to electrical excitation is affected by forearm posture, joint angle, or a combination of both factors. This study aimed to investigate the effects of forearm postures and elbow joint angles on the muscle torque and MMG signals. Measurements of the torque around the elbow and MMG of the biceps brachii (BB) muscle were conducted in 36 healthy subjects (age, 22.24 ± 2.94 years; height, 172 ± 0.5 cm; and weight, 67.01 ± 7.22 kg) using an in-house elbow flexion testbed and neuromuscular electrical stimulation (NMES) of the BB muscle. The BB muscle was stimulated while the forearm was positioned in the neutral, pronation, or supination positions. The elbow was flexed at angles of 10°, 30°, 60°, and 90°. The study analyzed the impact of the forearm posture(s) and elbow joint angle(s) on the root-mean-square value of the torque (${TQ}_{RMS}$). Subsequently, various MMG parameters, such as the root-mean-square value (${MMG}_{RMS}$), the mean power frequency (${MMG}_{MPF})$, and the median frequency (${MMG}_{MDF}$), were analyzed along the longitudinal, lateral, and transverse axes of the BB muscle fibers. The test–retest interclass correlation coefficient (ICC_21_) for the torque and MMG ranged from 0.522 to 0.828. Repeated-measure ANOVAs showed that the forearm posture and elbow flexion angle significantly influenced the ${TQ}_{RMS}$ (*p* < 0.05). Similarly, the ${MMG}_{RMS}$, ${MMG}_{MPF}$, and ${MMG}_{MDF}$ showed significant differences among all the postures and angles (*p* < 0.05). However, the combined main effect of the forearm posture and elbow joint angle was insignificant along the longitudinal axis (*p* > 0.05). The study also found that the ${MMG}_{RMS}$ and ${TQ}_{RMS}$ increased with increases in the joint angle from 10° to 60° and decreased at greater angles. However, during this investigation, the ${MMG}_{MPF}$ and ${MMG}_{MDF}$ exhibited a consistent decrease in response to increases in the joint angle for the lateral and transverse axes of the BB muscle. These findings suggest that the muscle contraction evoked by NMES may be influenced by the interplay between actin and myosin filaments, which are responsible for muscle contraction and are, in turn, influenced by the muscle length. Because restoring the function of limbs is a common goal in rehabilitation services, the use of MMG in the development of methods that may enable the real-time tracking of exact muscle dimensional changes and activation levels is imperative.

## 1. Introduction

The torque generated at a joint depends on the activation of the motor units in the muscle and on the mechanics of the muscle fibers and muscle–tendon units [1]. These variations have a significant effect on the muscle activity [2] and output torque. However, due to the increase in neurological impairments, neuromuscular electrical stimulation (NMES) has been found to be of clinical significance in functional rehabilitation. To further assess the effectiveness of NMES, it is required to understand the muscle’s activity, stimulus-elicited torque, and MMG responses to motor unit recruitment. The old standard, electromyography (EMG), is used to assess the torque and contractile properties of muscles but has limitations due to significant interference from electrical signals, artefacts, and skin impedance changes [3].

As a mechanical counterpart to EMG, with no interference from electromagnetic interference, mechanomyography (MMG) has been approved in the assessment of muscle torque [4]. MMG is generated by changes in the muscle shape and length that cause lateral oscillations of muscle fibers at the resonant frequency of the muscle and the dimensional changes of muscle fibers. These oscillations are quantified using the root mean square of the MMG amplitude (${MMG}_{RMS}$) and frequency domain parameters [5], such as the mean power frequency (${MMG}_{MPF})$ and median power frequency (${MMG}_{MDF}$).

Early studies defined the relationship between the ${MMG}_{RMS}$ and muscle length as a function of the joint angle [6]. However, when considering dynamic muscle activity, measuring the effects of a specific muscle becomes challenging at a joint. For example, the biceps brachii (BB), brachialis, brachioradialis muscles [7], and forearm muscles, such as the pronator tares and the flexor carpi radialis, contribute to the elbow joint torque. Consequently, the coactivation of these muscles can mask the behaviors of the BB muscle. A previous study showed that the forearm posture effectively impacts neural control, with the longest length of BB observed during pronation, followed by during the neutral and supination positions [8]. Acknowledging the influence of forearm posture on neuropathways could enhance the understanding of these torque and MMG findings. While research has highlighted the importance of MMG frequency and RMS parameters for slight muscle contractions [4], understanding of the muscle strength evaluation can be gained from biomechanical effects due to muscle size and morphology changing with forearm posture.

Study [9] found that MMG parameters below 20% MVC might correlate with sustained contraction in the slow-twitch muscle fibers. However, they exhibited coactivation of nearby muscles, which influenced the interpretation of the findings. Interestingly, NMES has demonstrated activation of targeted muscles [10] at levels up to 20% MVC [11], underscoring the need to explore how joint factors influence the muscle contraction and torque outcomes [12,13,14,15]. Specifically, the normalized ${MMG}_{RMS}$ value and knee extension torque increased with increases in the flexion angle [6]. This finding is important because a change in the joint angle is related to the biomechanical properties of the related muscle(s) [16,17], which affect the motor unit recruitment [11,12] and overlapping of cross-bridge elements [17,18]. In addition, voluntary contraction of the BB revealed a monotonic increase followed by a decrease in the ${MMG}_{RMS}$ with increases in the elbow joint angle [19]. Typically, ${MMG}_{RMS}$ decreases at 90% of the muscle length [20] and increases at other lengths. Therefore, this finding suggests that the ${MMG}_{RMS}$ reflects the power of the muscle [21], which is specific to the angle [6], and is influenced by the motor unit recruitment strategy [22,23]. A downshift in the ${MMG}_{MDF}$ with an increase in the elbow joint angle indicates the firing rate of motor units [15,18] at shorter muscle lengths.

As mentioned previously, the length and shape of the BB muscle vary with changes in elbow flexion, which makes understanding the muscle’s response to NMES important. However, although a recent study [19] investigated the relationship of the elbow joint angle with the ${TQ}_{RMS}$ and ${MMG}_{RMS}$, muscle behaviors with different forearm postures and different elbow joint angles have not been studied in NMES-evoked contractions. Furthermore, the significance of the ${MMG}_{RMS}$, ${MMG}_{MPF}$, and ${MMG}_{MDF}$ along the axes of muscle fibers has not been assessed. It is worth noting that the variation in the spectral responses of the muscle is influenced by muscle fibers during multiscale movement [24].

Given the importance of this article, we measured the influence of the forearm posture(s) and elbow joints angle(s) on ${TQ}_{RMS}$, ${MMG}_{RMS}$, ${MMG}_{MPF}$, and ${MMG}_{MDF}$ values during consistent NMES of the BB muscle. We hypothesized that: (1) The linear increase and decrease in ${TQ}_{RMS}\;and\;{MMG}_{RMS}$ with changes in the forearm position and elbow joint angle validate MMG as an alternative to complicated torque measurement in occupational activities [14] and reflect preceptive feedback useful in post-surgical monitoring of the muscle at specific elbow joint angles based on the affected length-dependent muscle afferent units [15]. (2) We also postulated that the ${MMG}_{MPF}$ and ${MMG}_{MDF}$ either remain consistent or exhibit a downward trend with increases in the elbow joint angle, indicating how muscles are activated [10], which is useful in the design of externally powered prosthesis when the MMG amplitude is inefficient.

The remainder of this paper is arranged as follows: Section 2 describes the acquisition of MMG and torque signals and the calculations of the MMG and torque parameters used for analysis; Section 3 describes the experimental results; Section 4 discusses the findings of this study; and the conclusion is provided in Section 5.

## 2. Materials and Methods

### 2.1. Subjects

Thirty-six healthy males (age, 22.24 ± 2.94 years; height 172 ± 0.5 cm; and weight, 67.01 ± 7.22 kg) participated in this study. The subjects had no history of neuromuscular disorders or surgical operations. Before participation, the subjects provided a written and signed informed consent after disclosure of the study’s purpose and procedures and completed a health history screening. The subjects were briefed about their rights to withdraw their participation at any time. Ethical approval of the experimental protocol (NMRR-20-2613-56796 (IIR)) was obtained from the Medical Research Ethics Committee of Malaysia in compliance with the principles outlined in the Declaration of Helsinki. The experiment was conducted at the laboratory at the Faculty of Electronics and Computer Engineering, Universiti Teknikal Malaysia Melaka (UTeM).

### 2.2. Experimental Protocol

The subjects visited the laboratory on three different occasions. Demographic data were recorded in the familiarization session, and all subjects completed a warmup protocol to familiarize themselves with performing maximum voluntary contraction (MVIC) of elbow flexion with the right hand at 90°. The MVIC exercise was repeated once the error between the two consecutive trials exceeded 5%. A minimum of 5 min of recovery was provided to avoid muscle fatigue.

Upon determining MVC, the participant was familiarized with the sensation of NMES intensity. The motor point was determined, and the magnitude of stimulation intensity was determined for the maximum comfort of the subject. The subjects whose BB muscle failed to provide about 15% of equivalent NMES were withdrawn from the study [2]. This value was obtained for most participants at a stimulation protocol of a frequency of 30 Hz, a pulse width of 110 µs, and a current amplitude of 30 mA, which induces elbow flexion torque without causing discomfort the participant. Thereafter, the subjects refrained from any strenuous muscle activity for at least 24 h before the next NMES experiment session.

**Figure 1:**
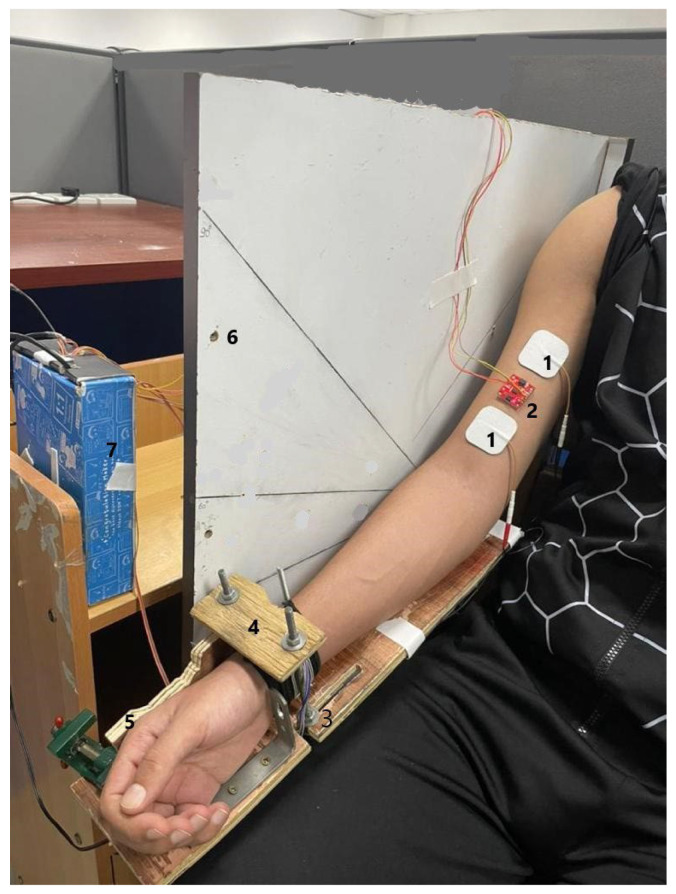
Raw acceleration and force data acquisition setup: (1) electrical stimulation electrodes, (2) MMG sensor, (3) adjustable elbow rest, (4) support for the force sensor (placed underneath), (5) a fixture for the hand/wrist posture, (6) a fixture for the adjustable elbow joint angle, and (7) force and acceleration data acquisition device.

During the experiment, the forearm was secured in either the neutral, pronation, or supination position and fixed to a rotating custom-made wooden arm at the desired elbow joint angle (Figure 1). Following the guidelines of the International Society of Electromyography and Kinesiology (ISEK), a self-adhesive electrode (4 cm × 4 cm, Hercusense TENS/EMS, V2U Healthcare Pte. Ltd., Singapore) was placed on the motor point, and the distal electrode was placed at the other end of the BB muscle belly. The stimulation intensity was delivered by electrical muscle stimulation (EMS 7500, V2U Healthcare Pte. Ltd., Singapore). An indelible marker was utilized to ensure the consistent positioning of the stimulation electrodes throughout the experimental sessions. The muscle belly was located through palpation with the elbow flexed at 90° to ensure proper fixation of the MMG sensor. NMES was applied to the BB muscle while the subject maintained different forearm postures and elbow joint angles (10°, 30°, 60°, and 90°), measured using a digital goniometer.

To ensure consistency, the lever arm of the forearm was kept the same for all the participants using adjustable table vices and screws, as shown in Figure 1. The arm posture and elbow joint angle were maintained and monitored by two assistants present at the site. To ensure full recovery of the muscle, the participants were allowed to rest for at least 10 min between two postures or angles and 5 min between trials. Each trial lasted 30 s.

### 2.3. Data Acquisition and Signal Processing

Acceleration and muscle force data were simultaneously recorded by a customized LabVIEW program (NI LabVIEW 2021 (64 bit)) at a sampling rate of 1 kHz. The Arduino Uno R3 Microcontroller was used to interface the acceleration and force transducers. Acceleration data were detected using an ADXL-313 (weight < 2.6 g, low power, high resolution (up to 13 bits)), a 3-axis accelerometer capable of measuring up to ± 4 g (SparkFun, Niwot, CO, USA). An ADXL-313 sensor was attached to the muscle belly using adhesive tape (3MTM VHBTM 4920, St. Paul, MN, USA). The force was measured using a force transducer (FS2050 Compression LC1500 GRAM, TE Connectivity, Schaffhausen, Switzerland), and the lever measured from the olecranon process to styloid process of the ulna.

The dataset included three axes of acceleration signals and one torque value for each posture and angle. The power spectrum of MMG was found below 100 Hz [25]. The MMG data were obtained by filtering acceleration data using a fourth-order Butterworth band-pass filter of 5–100 Hz to eliminate the adverse artefacts from electrical cabling and body movement [26]. The torque data were filtered using a 4th-order Butterworth low-pass filter with a cutoff frequency of 5 Hz [27].

**Figure 2:**
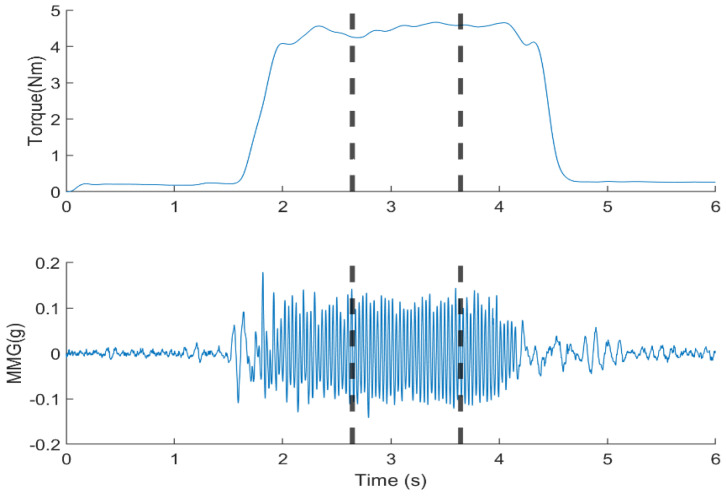
Filtered elbow joint torque and MMG signal from the BB. The data from the middle 1 s, bounded by the dotted lines, were used for the calculation of ${TQ}_{RMS}$, ${MMG}_{RMS}$, ${MMG}_{MPF}$ and ${MMG}_{MDF}$.

For analysis, the middle 1 s plateau of the torque and MMG signals [6] was determined using a moving window of 1000 ms at a threshold of 20% of the maximum muscle contraction above the baseline. This selection aimed to extract data with no effect on the onset of torque development at the beginning of muscle contraction and the offset at the start of muscle relaxation (Figure 2). A 512-point Short-Time Fourier Transform (STFT) with 50% window overlap was used to compute the MPF and MDF from the MMG signals. Thereafter, the ${TQ}_{RMS}$, ${MMG}_{RMS}$, ${MMG}_{MPF}$, and ${MMG}_{MDF}$ were calculated. Subsequent analysis was performed using a 100-millisecond window with 50% overlap. The ${TQ}_{RMS}$, ${MMG}_{RMS}$, ${MMG}_{MPF}$, and ${MMG}_{MDF}$ were then normalized to their respective maximum levels among the twelve conditions (four elbow joint angles and three forearm postures) [2]. MATLAB^®^ 2021b (Apple Hill Drive, Natick, MA, USA) was used for signal processing.

### 2.4. Statistical Analysis

The Shapiro–Wilk test was used to examine the normality of the distribution of ${TQ}_{RMS}$, ${MMG}_{RMS}$, ${MMG}_{MPF}$, and ${MMG}_{MPF}$ values obtained along the longitudinal, lateral, and transverse axes of the BB muscle fibers. One-way analysis of variance (ANOVA) with repeated measures was performed to evaluate the effect of the forearm posture on the torque and MMG parameters. Similarly, the effect of the elbow joint angle on the elbow flexion torque was assessed. Two-way analysis of variance (ANOVA) with repeated measures was utilized to examine the combined effects of the posture and elbow joint angle on torque and MMG variables. The Greenhouse–Geisser correction factor was applied whenever the sphericity assumption was violated. A Bonferroni adjustment was used as the post hoc test to assess the differences. The differences in the investigated variables were significant if *p* < 0.05. The reliability (ICC_21_) of the ${TQ}_{RMS}$ and ${MMG}_{RMS}$ measurement was assessed as excellent (>0.9), good (0.75–0.9), moderate (0.5–0.75), or poor (<0.05) [28]. All variables are presented as the normalized means (SDs) of the measurements. All the statistical tests were conducted using IBM SPSS 25.0 (SPSS Inc., Chicago, IL, USA).

## 3. Results

The reliability measured outcomes (ICC_2,1_) ranged from 0.688 to 0.823 for ${TQ}_{RMS}$, and from 0.522 to 0.761 for ${MMG}_{RMS}$ for all investigated elbow joint angles and forearm postures. The CV% exceeded 10% at 60° only for the neutral position. The minimum detectable change (MDC) ranged from 0.265 to 0.424. A paired-sample *t*-test revealed a non-significant difference for ${TQ}_{RMS}$ and ${MMG}_{RMS}$ across all testing sessions (*p* > 0.05).

### 3.1. Effect of the Forearm Posture and Joint Angle on Torque

**Figure 3:**
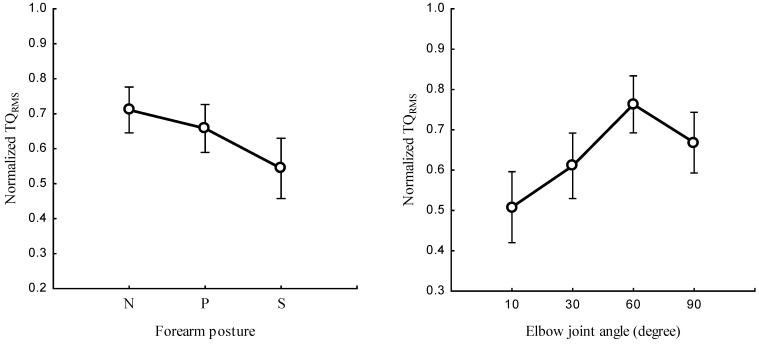
Normalized ${TQ}_{RMS}$ at neutral (N), pronation (P), and supination (S) postures of the forearm (**left**) and the elbow flexion at 10°, 30°, 60°, and 90° (**right**).

**Figure 4:**
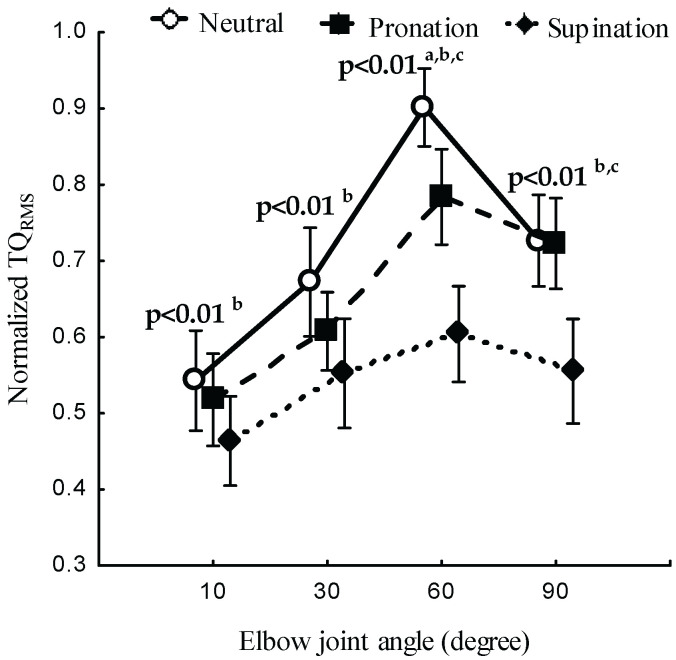
Normalized ${TQ}_{RMS}$ at the neutral, pronation, and supination positions of the forearm and at elbow joint angles of 10°, 30°, 60°, and 90°. The statistical significance between posture conditions are indicated (a—neutral and pronation, b—neutral and supination, c—pronation and supination).

Figure 3 and Table 1 present the relationship between the forearm posture, elbow joint angle, and ${TQ}_{RMS}$. The ${TQ}_{RMS}$ in the neutral position was higher than the values obtained in the pronation and supination positions (*p* < 0.05). The forearm posture had a significant effect on the ${TQ}_{RMS}$ (*p* < 0.05) at all elbow joint angles. The joint angle was found to have a significant effect on the normalized ${TQ}_{RMS}$ in the neutral (*p* < 0.05), pronation (*p* < 0.05), and supination (*p* < 0.05) positions. The forearm posture and elbow joint angle had a significant combined main effect on the ${TQ}_{RMS}$ (*p* < 0.05; Figure 4). The post hoc test revealed a significant ${TQ}_{RMS}$ (*p* < 0.05) at all angles between the neutral and supination position and among all postures at 60°.

### 3.2. Effect of the Elbow Joint Angle and Forearm Posture on the MMG Responses to NMES

#### 3.2.1. MMG Amplitude

**Figure 5:**
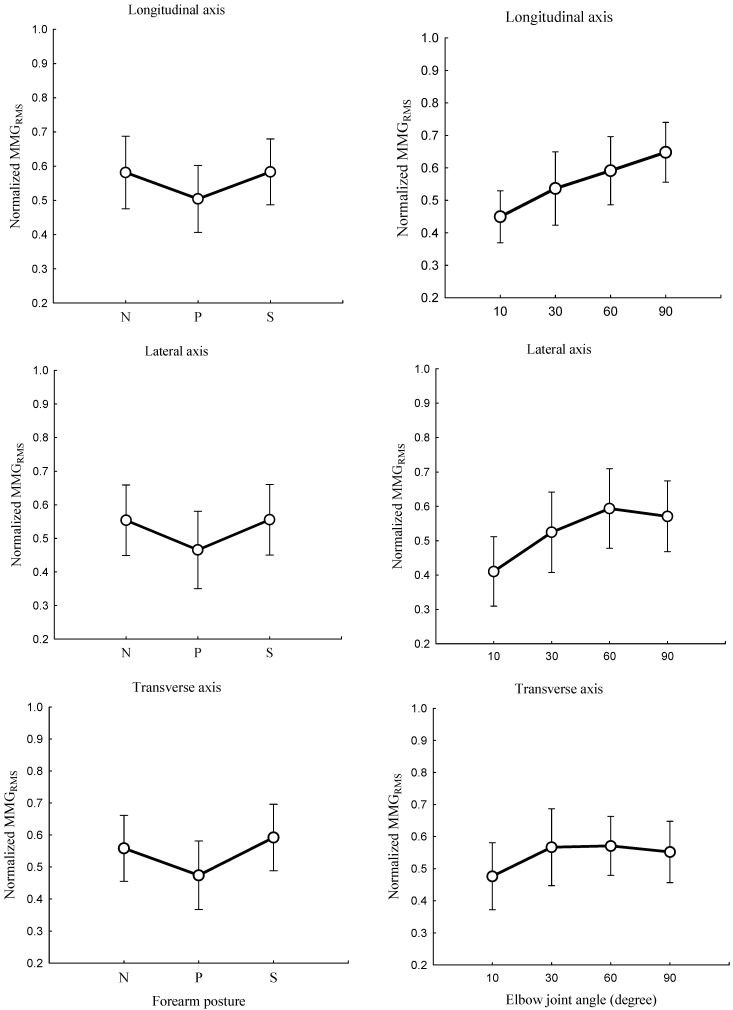
Behaviors of the normalized ${MMG}_{RMS}$ along the longitudinal, lateral, and transverse axes of the BB muscle fibers at the neutral (N), pronation (P), and supination (S) positions (**left**) and elbow joint at 10°, 30°, 60°, and 90° (**right**).

**Figure 6:**
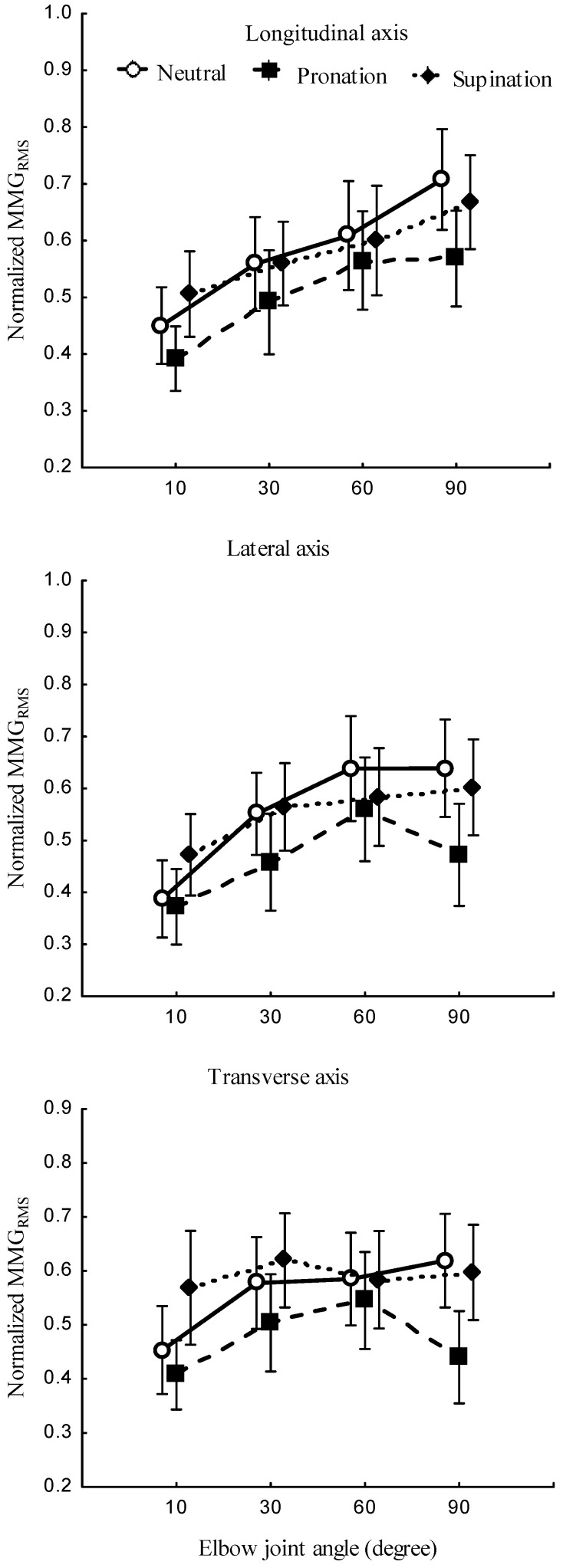
${MMG}_{RMS}$ along the longitudinal, lateral, and transverse axes of the BB muscle with the forearm in neutral, pronation, and supination positions and the elbow joint at 10°, 30°, 60°, and 90°.

The relationships between the forearm posture and the ${MMG}_{RMS}$ (Figure 5, left) and the elbow joint angle and the ${MMG}_{RMS}$ (Figure 5, right) and the main effect of the forearm posture and elbow joint angle on the ${MMG}_{RMS}$ (Figure 6) were examined along the longitudinal, lateral, and transverse axes of BB muscle fibers.

The forearm posture was found to have a significant effect on the normalized ${MMG}_{RMS}$ along the longitudinal axis (*p* < 0.05), the lateral axis (*p* < 0.05), and the transverse axis (*p* < 0.05). The elbow joint angle had significant effects on the normalized ${MMG}_{RMS}$ along the longitudinal (*p* < 0.05), lateral (*p* < 0.05), and transverse (*p* < 0.05) axes of BB muscle fibers. The interaction between the forearm posture and elbow joint angle was found to have no significant main effects on the ${MMG}_{RMS}$ along the longitudinal (*p* > 0.05), lateral (*p* > 0.05), and transverse axes (*p* > 0.05) of BB muscle fibers.

#### 3.2.2. MMG Frequency

**Figure 7:**
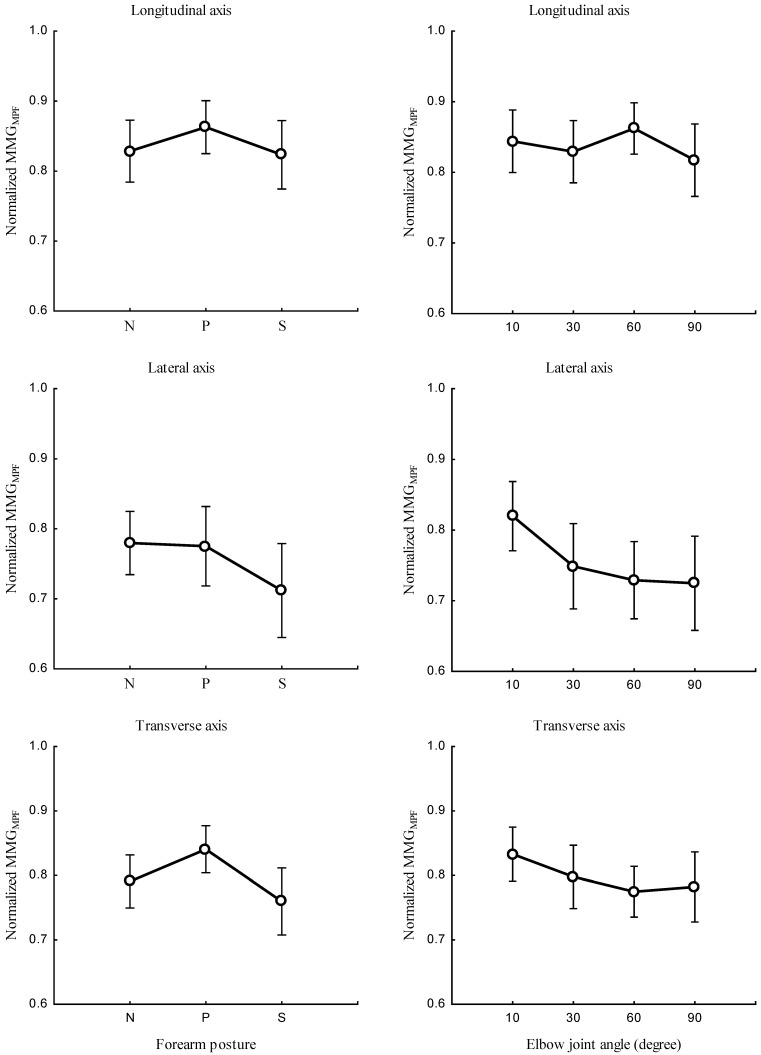
Relationship between ${MMG}_{MPF}$ along the longitudinal, lateral, and transverse axes of the BB muscle fibers at the neutral (N), pronation (P), and supination (S) (**left**) positions, and elbow joint at 10°, 30°, 60°, and 90° (**right**).

**Figure 8:**
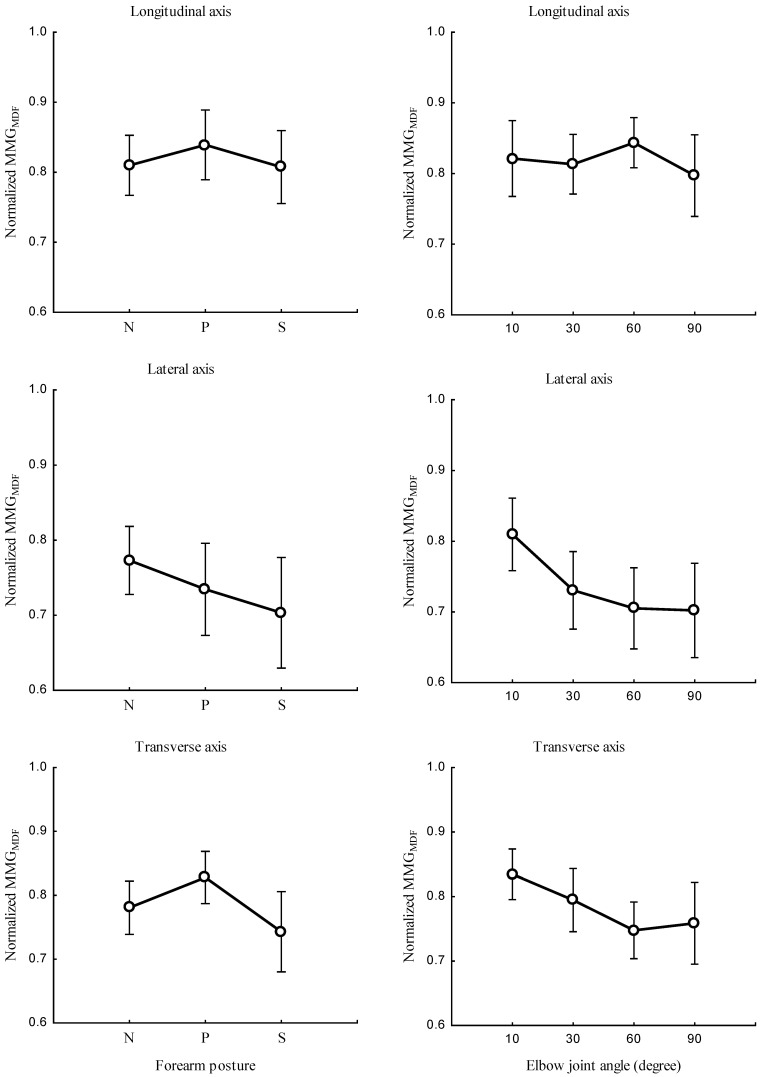
Behaviors of ${MMG}_{MDF}$ along the longitudinal, lateral, and transverse axes of the BB muscle fibers at the neutral (N), pronation (P), and supination (S) positions (**left**) and at elbow joint angles of 10°, 30°, 60°, and 90° (**right**).

**Figure 9:**
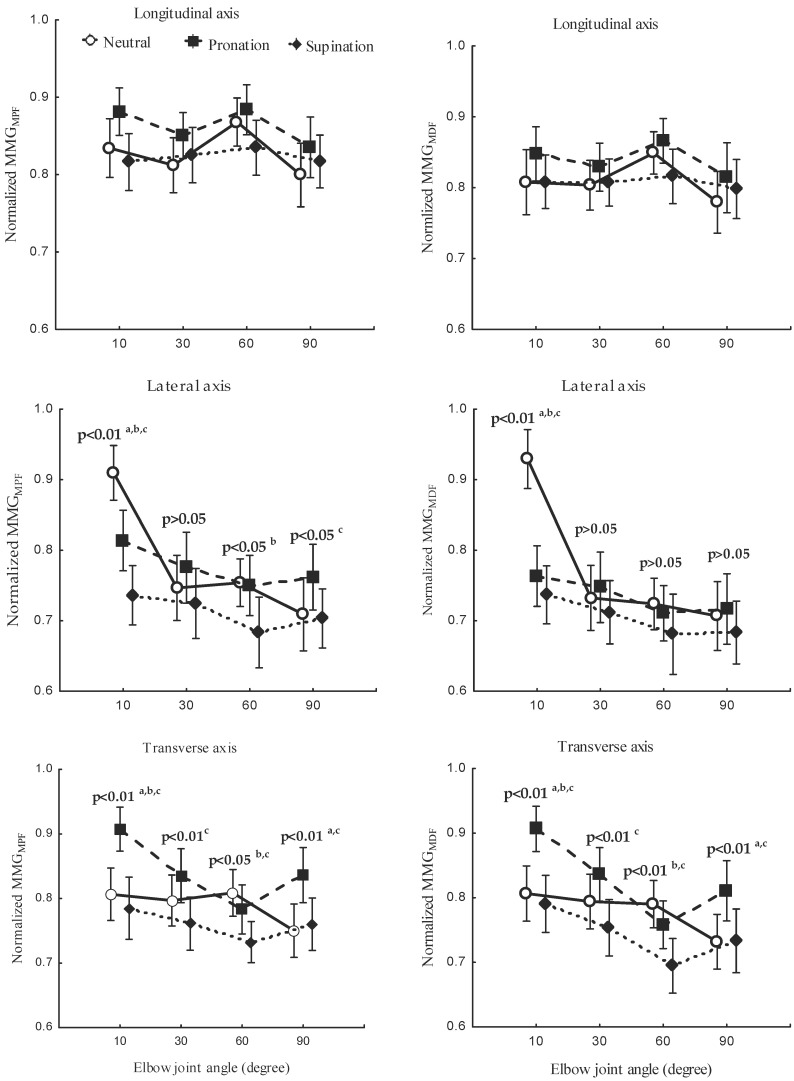
Behaviors of ${MMG}_{MPF}$ (**left**) and ${MMG}_{MDF}$ (**right**) along the longitudinal, lateral, and transverse axes of the BB muscle fibers at the neutral, pronation, and supination positions (**left**) and with the elbow joint at 10°, 30°, 60°, and 90° (**right**). The statistical significance between postures conditions are indicated (a—neutral and pronation, b—neutral and supination, c—pronation and supination).

The study investigated the correlation between the forearm posture and both ${MMG}_{MPF}$ (Figure 7, left), and ${MMG}_{MDF}$ (Figure 8, left) as well as the relationship between elbow joint angle and both ${MMG}_{MPF}$ (Figure 7, right), and ${MMG}_{MDF}$ (Figure 8, right). Measurements were taken along the longitudinal, lateral, and transverse axes of BB muscle fibers. The main effect of the interaction between the forearm posture and elbow joint angles on ${MMG}_{MPF}$ (Figure 9) left as well as on ${MMG}_{MDF}$ (Figure 9) right, were assessed along the longitudinal, lateral, and transverse axes of BB muscle fibers.

The forearm posture was found to have significant effects on the normalized ${MMG}_{MPF}$ and ${MMG}_{MDF}$ along the longitudinal axis (*p* < 0.05), the lateral axis (*p* < 0.05), and the transverse axis (*p* < 0.05). The elbow joint angle had significant effects on the normalized ${MMG}_{MPF}$ and ${MMG}_{MDF}$ along the longitudinal axis (*p* < 0.05), the lateral axis (*p* < 0.05), and the transverse axis (*p* < 0.05). The interaction between the forearm posture and elbow joint angle had insignificant effects on the ${MMG}_{MPF}$ and ${MMG}_{MDF}$ along the longitudinal axis (*p* > 0.05). Along the lateral and transverse axes, the interaction between the forearm posture and elbow joint angle had a significant effect on the ${MMG}_{MPF}$ and ${MMG}_{MDF}$ (*p* < 0.05). A significant difference in ${MMG}_{MPF}$ and ${MMG}_{MDF}$ (*p* < 0.05) was observed among all postures at 10° along the lateral and transverse axes. Both ${MMG}_{MPF}$ and ${MMG}_{MDF}$ were found to be insignificant between the neutral and pronation position (*p* < 0.05) and among all posture combinations at 30°, 60°.

## 4. Discussion

The results of this study indicate that the reliability of ${TQ}_{RMS}$ and ${MMG}_{RMS}$ parameters ranged from moderate to good, which demonstrates that MMG can reflect the biomechanical properties of muscles [29] and give further insights in the investigation of muscle physiology. This study indicates that (1) the forearm posture and (2) elbow joint angle influenced the neural excitation of the biceps brachii remarkably in muscle strength assessment and neuromuscular rehabilitation during NMES.

### 4.1. Effect of the Forearm Posture and Elbow Joint Angle on Torque

The results show that changing the forearm position significantly affected the normalized ${TQ}_{RMS}$ (Figure 3). This finding suggests that variation in the forearm posture leads to variation in the muscle length, shape, and size which, in turn, influences the spinal excitability of the BB muscle [8]. This effect may affect the responsiveness of the BB muscle to NMES. Therefore, these results validate the hypothesis that the influence of the forearm posture on the ${TQ}_{RMS}$ is associated with alterations in muscle morphology, which cause the results to deviate from the expected outcomes. Specifically, while a consistent NMES intensity is maintained, a decreased ${TQ}_{RMS}$ at the supination position might be caused by the reduction in the current density with increased depth of the BB muscle, thus compromising the level of recruitment of deeper muscle fibers [30].

Similar to the findings obtained in previous studies, the present research found that the elbow joint angle had a significant effect on the ${TQ}_{RMS}$ [31,32]. Across 10°, 30°, and 90° angles, there was a significant difference between the neutral and supination position and among all postures at 60°. These behaviors are caused by the overlap between the actin and myosin that disrupts the formation of a cross-bridge connection and hinders the force production [33]. With increases in the elbow flexion angle beyond 60°, the ${TQ}_{RMS}$ exhibited a downward shift. These results indicate that the resting length produces more cross-bridge interactions between actin and myosin filaments around 60°. The reduction is attributed to mechanical interaction among neighboring actin filaments at shorter muscle length and the stretching of actin and myosin at longer muscle length, which, in turn, influence the NMES-evoked ${TQ}_{RMS}$ [34]. Additionally, because the BB originates from the radial tuberosity, elbow flexion leads to a reduction in the length of the BB due to variation in the moment arms. These changes are associated with the level of neural excitation [35] and an altered fiber-type composition [17,21,29,30,31]. NMES shows greater torque at intermediate elbow joint angles [36], which indicates variation in the morphological adaptations of muscle tendons [37] and changes in the sarcomere length [19,33].

In this study, a consistent NMES intensity was employed. However, during elbow flexion, some of the deeper motor units were not activated. Consequently, the observed downward trend in torque was influenced by the reduction in the motor units activated per electrode site [30]. This finding indicates that the changes in the muscle depth due to variations in the forearm posture and elbow joint angle impact the electrical excitation of the muscle. An early study showed that the motor point of the biceps shifts from 1.5 cm to 2 cm over a range of 80° [38]; the electrode size of 4 cm × 4 cm used in our experiment was sufficient for the electrode to remain fixed at the threshold location of the motor point. Thus, the variations in the output measurement (${TQ}_{RMS}$) were strongly influenced by changes in the biomechanical and mechanical characteristics of the BB muscle.

This research is the first to highlight NMES-evoked torque with variations in the forearm posture and elbow joint angle at below 15% equivalent MVC. These findings suggest that the lever arm, joint structure, and myosin were affected and thus altered the motor output, which is dependent on the length. This finding was observed because the muscle length modulates the local circuit neurons [8], which influence the responsiveness of the muscle to NMES, a necessary feature in therapeutic rehabilitation. Additionally, previous studies revealed that 20% torque evoked by NMES provokes a higher degree of muscle activation compared with equivalent 20% MVC torque in lower limb muscles [11]. The 15% NMES-evoked torque in this study supports the belief that it is beneficial to restore the muscle tissue recovery of injured muscles and to carry out post-surgical recovery monitoring [2]. However, the forearm posture influences the spinal pathways, though this finding was not evident in MVC. Further investigation should focus on the optimum protocol for achieving an MVC equivalent to 15% of NMES with changes in the muscle length.

The inter-individual responses to NMES were found to be influenced by body fat, BMI, and sex. Although our experiment included subjects within a normal, healthy BMI range, it is worth noting that the upper arm circumference impacted the responsiveness of the muscle to NMES [39]. Therefore, understanding the effects of anthropometric measures coupled with NMES on muscle strength could serve as a valuable direction for future clinical studies.

### 4.2. Effect of the Forearm Posture and Elbow Joint Angle on the MMG Amplitude

An alteration in the forearm posture influences the electrophysiological properties of the BB muscle [40]. These changes are associated with inhibitory and excitatory circuits of the spinal pathways. During elbow flexion accompanied by forearm rotation, the dimensional changes of muscle fibers lead to variations in their firing rates, which are reflected in the form of MMG signals [41].

In this study, the forearm posture was found to have a significant effect on the ${MMG}_{RMS}$ along the longitudinal, lateral, and transverse axes. These findings indicate that the forearm posture causes a change in the behaviors of the neural connections that carry signals to the BB muscle [42]. The insignificant difference in the ${MMG}_{RMS}$ at 30° and 60° can be attributed to insufficient excitability in deeper muscle fibers due to a muscle length shorter than the resting length of the BB muscle [8]. This is useful in correcting postural balance and optimizing the neural output in mobility rehabilitation.

Across the investigated elbow joint angles, the pronation posture consistently yielded the lowest ${MMG}_{RMS}\;$(Figure 5, left, and Figure 6). This finding is consistent with a previous study [7] and can be attributed to the inhibition of the activation of BB muscle fibers under the same synaptic connection with the spinal tracks of the brachioradialis muscle. NMES experiments have shown that isolating the BB from the brachialis and brachioradialis muscles may lead to an additive level of excitation which can be decreased in the pronation position [7]. This finding may be caused by the muscle–tendon complex, which experiences greater stiffness at higher torque output (Figure 3, left, and Figure 4). Furthermore, the magnitude of each of the three axes varied across the forearm postures. These findings suggest that the forearm can produce reach information on the muscle performance outcome.

An increase in the elbow joint angle induced an increase in the ${MMG}_{RMS}$. This observation complies with the approach taken by Barry [43], who found greater MMG amplitudes for an electrically stimulated gastrocnemius muscle with shorter muscle lengths. As supported by previous research [30], the recruitment of motor units in deeper muscle(s) depends on the number of fibers under the stimulation electrodes. Furthermore, the filtering capacity of the tissue between the acceleration sensor and recruited muscle fibers increases with increases in the elbow flexion angle [44]. This study also showed that the ${MMG}_{RMS}$ along the lateral and transverse axes increased as the elbow flexion angle increased from 10° to 30° and decreased as the angle increased from 60° to greater values (Figure 5, right). As also observed in a previous study [20], the MMG amplitude increased at 90% of the muscle length and decreased when the muscle length was higher or lower than 90%. This implies that MMG amplitude can reflect the angle at which the muscle strength is optimized when the torque measurement is complicated, such as in neurologic amputees.

The significant differences in the elbow joint along the longitudinal, lateral, and transverse axes can be attributed to the dynamic properties of the muscle, also influenced by the viscosity, thickness, and stiffness, all of which might be varied with changes in the muscle length. Hence, the finding evidently shows that the difference in the ${MMG}_{RMS}$ along the lateral axis mirrors the resonance frequency of the fiber, and the difference along the transverse axis reflects the longitudinal stiffness [43].

### 4.3. Effect of the Forearm Posture and Elbow Joint Angle on the MMG Frequency

This study found that an increase in the elbow joint angle was associated with a decrease in the ${MMG}_{MPF}$. Evidence of these findings shows that when a muscle is selectively isolated, the decline in the ${MMG}_{MPF}$ originates from the lengthening or shortening of the sarcomere outside the nominal dimensions of actin and myosin. Additionally, an increase in elbow flexion causes an increase in the muscle diameter, which influences the insufficient overlap of actin and myosin filaments [45]. This finding agrees with the research reported by Frangioni, who found that the mean power frequency increased with increases in the muscle length of the gastrocnemius muscle of a frog under electrical stimulation [46]. Furthermore, studies of voluntary contraction claimed a significant decrease in the ${MMG}_{MPF}$ at shorter muscle lengths [47]. Together, these earlier findings and those obtained in this study demonstrate that the MMG response is independent of the firing patterns but dependent on the contraction and relaxation time properties of the muscle fibers [20].

The decreased ${MMG}_{MDF}$ found in this study was influenced by the muscle length [41]. The patterns found for the ${MMG}_{MDF}$ were higher at greater muscle lengths [48]. A decrease in the ${MMG}_{MDF}$ with an increase in the elbow joint angle indicates motor unit synchronization, which reportedly reduces the median frequency of the muscle [49]. The difference in the patterns found for ${MMG}_{MDF}$ along the longitudinal, lateral, and transverse axes indicates that the levels of activation of muscle fibers vary among different elbow angles and forearm postures. Specifically, the ${MMG}_{MPF}$ and ${MMG}_{MDF}$ along the longitudinal axis (Figure 7, right, and Figure 8, right) increased and decreased with an increase in the ${TQ}_{RMS}$. This finding indicates that the frequency features of MMG signals measured along the longitudinal axis can mirror the patterns of NMES-evoked torque. Conversely, the ${MMG}_{MPF}$ and ${MMG}_{MDF}$ along the lateral and transverse axes exhibited a meaningful decrease, which was different to the results found for the ${TQ}_{RMS}$ (Figure 9). This non-linear feature is caused by the muscle architecture, tendon units, and sensory feedback at the muscle spindles. Additionally, the decline in both the ${MMG}_{MPF}$ and ${MMG}_{MDF}$ indicates the discharge rate of Ia afferent inputs, which have been shown to vary with changes in the resting length of the muscle. The lack of significant difference found between the neutral and the pronation position showed the notable muscle fiber recruitment with a longer muscle [3,8]. The non-linear relationship of the MMG frequency features reveals that spectral parameters are good candidates for limb torque estimation and could be further explored in the future. Furthermore, ${MMG}_{MPF}$, ${MMG}_{MDF}$ and ${MMG}_{RMS}$ in this study outperformed the variability in multiple-degree-of-freedom activities [50] and the performance of EMG in electric hand prothesis for the limb-imputed population [51].

The limitation of the current study is that the analysis was centered on the response of the BB muscle. For future investigation, the brachioradialis and brachialis muscles should be included. The findings were limited to a constant intensity of NMES and a long span of joint angles. Hence, varying the intensity of NMES could offer insights into neuromuscular efficiency, which is a proxy for gauging the effectiveness of rehabilitation exercises in future research.

## 5. Conclusions

The study evaluated the relationship between elbow joint angles, forearm posture, and NMES-elicited muscle contraction in normal, healthy subjects and showed reliable MMG and torque. These findings suggest that MMG is a proxy tool for non-invasive assessment of NMES-evoked contraction in the BB. ${MMG}_{RMS}$, ${MMG}_{MPF}$ and ${MMG}_{MDF}$ along the longitudinal axis showed a monotonic increase with the elbow joint angles. Hence, these parameters should be used to monitor the muscle activation in daily occupational tasks. ${MMG}_{MPF}$ and ${MMG}_{MDF}$ along the lateral and transverse axis decreased at shorter muscle length (Figure 9). ${TQ}_{RMS}$ parameters used in this study linearly increased from 10° to 60° and decreased beyond 60°. Thus, there is a specific joint angle at which the NMES-evoked torque is at its maximum. These findings are consistent with the reported results of the previous research [20], where the magnitude of muscle vibration increased with nominal muscle length to 90% and declined at other muscle lengths. Therefore, under the condition of joint angle, MMG signals are sensitive to contractile elements of the muscle; thus, they should be useful for characterizing the peripheral and central response to NMES. The reliability of MMG and torque provides the useful insight that MMG signals could be used to assess the function of skeletal muscles where expensive modalities are not possible. Typically, MMG parameters are evidence to be used by physical therapy practitioners which require the monitoring of post-injury and post-operative functionality [10]; thus, there remains scope for future investigation of other elbow flexor muscles.

## Figures and Tables

**Table 1:** Elicited elbow flexion ${TQ}_{RMS}$ measured at the elbow joint angle of 10°, 30°, 60°, and 90° in the Neutral (N), pronation (P), and supination (S) positions.

Elbow Joint Angle		10°		30°		60°		90°	
**Forearm** **Posture**		**Mean ± SD**		**Mean ± SD**		**Mean ± SD**		**Mean ± SD**	
	N	0.4516	0.1775	0.5707	0.1990	0.7416	0.1839	0.6029	0.2051
	P	0.4325	0.1724	0.5108	0.1700	0.6500	0.1704	0.5957	0.1797
	S	0.4002	0.1678	0.4524	0.1970	0.5041	0.2033	0.4639	0.1939

## Data Availability

Data are available upon reasonable request.
